# Use of drinkers by finisher pigs depend on drinker location, pig age, time of day, stocking density and tail damage

**DOI:** 10.3389/fvets.2022.1029803

**Published:** 2022-11-25

**Authors:** Mona Lilian Vestbjerg Larsen, Lene Juul Pedersen

**Affiliations:** Department of Animal and Veterinary Sciences, Aarhus University, Tjele, Denmark

**Keywords:** finisher pigs, animal welfare, drinker use, drinking behavior, abnormal behavior, tail damage, space allowance

## Abstract

Water is a vital nutrient for mammals, including the pig. Despite this, the use of drinkers and water have not yet been explicitly quantified across the finisher period. The current study aimed at gaining greater insight into finisher pigs' drinker use and its relation to drinker location, age, time of day, stocking density, enrichment provision and tail damage. The experiment included 110 pens of finisher pigs over a 9-week period, with two drinker cups per pen. Pens had a stocking density of either 0.73 m^2^/pig (*n* = 54 pens, 18 pigs per pen) or 1.21 m^2^/pig (*n* = 56 pens, 11 pigs per pen), were either provided with straw (*n* = 54, 150 g per pig and day) or not (*n* = 56), and had pigs with either undocked (*n* = 50) or docked tails (*n* = 60). Drinker use was recorded automatically by water-flow meters and summed to L and number of activations per hour and pig. Pens never experiencing a tail damage event (at least one pig in the pen with a bleeding tail) were used to investigate the normal drinker use of finisher pigs (*n* = 56). The water use of pigs increased from 3.7 to 8.2 L per pig and day during the 9 weeks, and this increase was mainly seen during the two large peaks of the diurnal pattern within the pigs' active period (06:00–18:00 h). No such increase was seen in the activation frequency at average 50 activations per pig and day. A decrease in stocking density increased both water use and activation frequency during the active period, suggesting that pigs at the standard space allowance and pig:drinker ratio could be restricted in their access to the drinking cups. The pigs also seemed to prefer to use the drinking cup closest to the feeder. Water use and activation frequency did not change the last 3 days prior to an event of tail damage, but general differences were seen between pens with and without a tail damage event. The current results may explain the success of previous studies in classifying tail damage pens from pens without tail damage using sensor data on drinker use.

## Introduction

Water is a vital, and probably the most important, nutrient for mammals, including the pig. As stated in the EU Council Directive 2008/120/EC on laying down minimum standards for the protection of pigs: all pigs over 2 weeks of age must have permanent access to a sufficient quantity of fresh water. Furthermore, the freedom from thirst is part of the Five Freedoms of animal welfare (1) and is included as an indicator in the assessment of animal welfare, such as the Welfare Quality^®^ protocol (2). Despite access to water is important, only few studies have investigated the water use of pigs [e.g., (3–6)], compared to other parts of the production such as feeding and access to enrichment. This lack of attention may be explained by that water seems to always be within reach of indoor-housed pigs. However, drinkers or water may be inaccessible due to low water flow, failures in the drinkers, contamination of drinkers by for example feces or inadequate space in the pen resulting in pigs being forced to lie in front of the drinker. Although such resource competition may be compensated by a change in the diurnal drinking pattern (3, 4), it may also cause stress and frustration in the pigs. Thus, not only pen-level water use may be important, but also drinker-level. To the authors knowledge, no study has yet explicitly quantified the water use of pigs across the entire finisher period, neither on pen-level nor drinker-level. However, the water use has been modeled during this period (7, 8) and a recent study did record water use of pigs throughout this period with a focus on the effect of group size and enrichment (6). Beside the intrinsic value of water for pigs, the drinking behavior may also have diagnostic value in relation to disease and other undesirable events, as found by multiple studies in relation to diarrhea, pen fouling and tail damage (7–10). However, how the drinking behavior of pigs change prior to such events has yet to be elucidated.

The current study aimed at greater insight into pigs' normal use of drinkers across the finisher period including water use and drinker activation frequency. This was achieved by (1) modeling the diurnal pattern and age trend on pen level, (2) relating pen level use of drinkers to tail docking, straw provision, and stocking density, and (3) quantitatively describing the diurnal pattern and age trend at drinker level (drinker location). The current study further aimed at greater insight into an applied case of pigs' drinker use in relation to tail damage. Based on previous studies, we hypothesized that the water use of pigs would increase with age and would show a diurnal pattern with one or more peaks during the active hours of the day. Furthermore, we hypothesized that stocking density would change the diurnal pattern in water use, with a higher stocking density resulting in higher water use during the less active hours of the day. Based on the success of previously developed prediction models of tail damage in finisher pigs, we also hypothesized a difference in water use between pens developing and not developing tail damage.

## Materials and methods

The present study was conducted from 2015 to 2016 in accordance with a protocol approved by the Danish Animal Experiments Inspectorate (Journal no. 2015-15-0201-00593). Further details about the experimental setup can be found in Larsen et al. (11) who studied the same animals and where the purpose of the study was to investigate risk factors to tail biting. The sensor data used in the current study were also used in the development of a prediction algortithm for tail damage in finisher pigs (7).

### Animals, housing, and management

**Figure 1:**
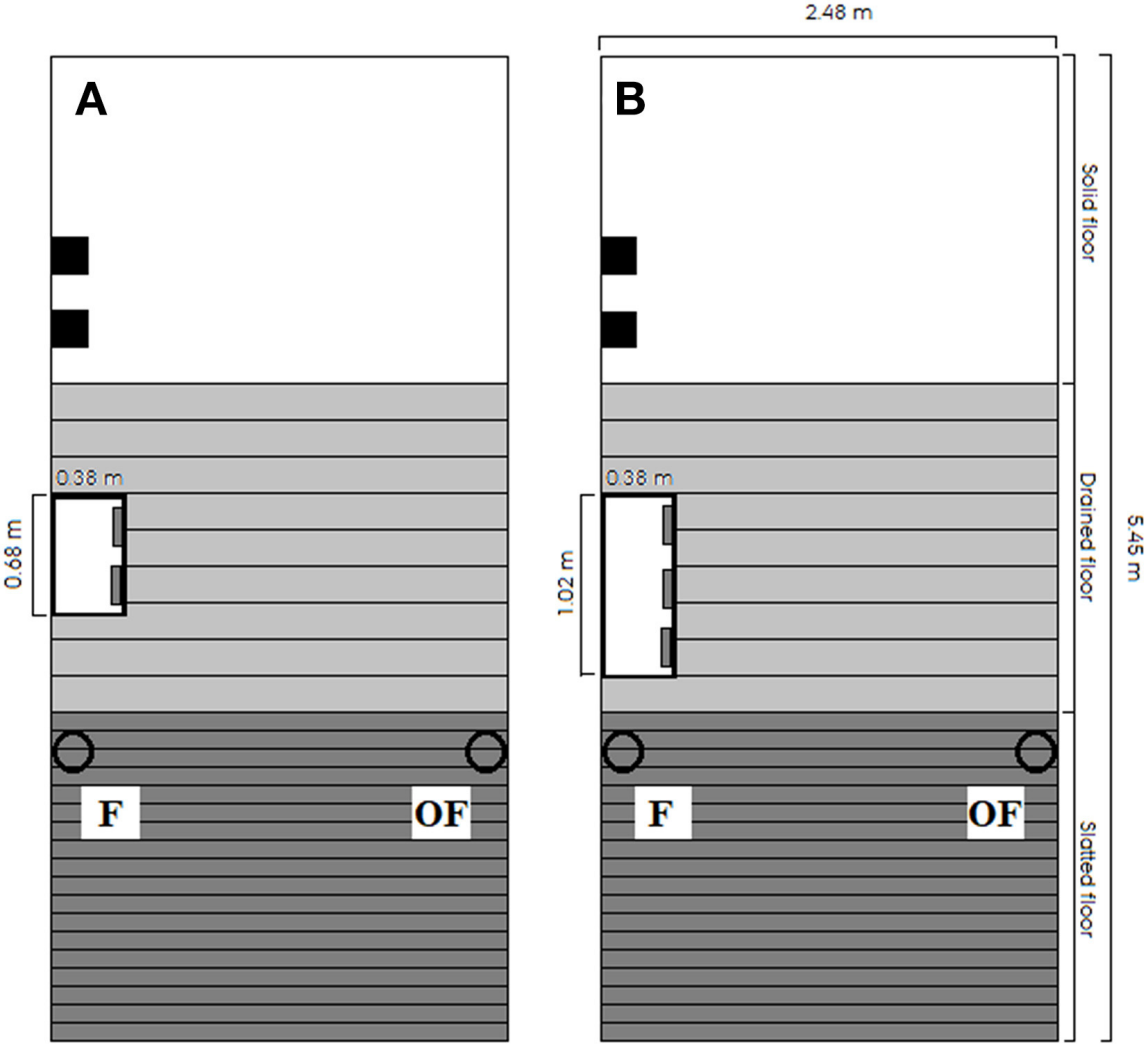
Drawing of pen dimension and design for **(A)** pens with a stocking density of 1.21 m^2^/pig (11 pigs) and **(B)** pens with a stocking density of 0.73 m^2^/pig (18 pigs). The white rectangle represents the feeder, and the solid black squares represent two wooden beams in separate vertical racks. The hollow black circles represent drinking cups, and all pens included a drinking cup on the same side of the pen as the feeder (F) and a drinking cup on the opposite side of the pen as the feeder (OF). All pens had the same dimensions.

The study was conducted in the experimental pig facilities at the Department of Animal and Veterinary Sciences, Aarhus University, Denmark, from June 2015 to November 2016 across four batches of finisher pigs. Each batch lasted 9 weeks. The study included 112 finisher pens (batches 1, 3 and 4: 32 pens; batch 2: 16 pens) with a total of 1,624 finisher pigs with an average introduction weight of 31.6 ± 6.6 kg. All pigs were purchased from the same Danish production herd and were all bred from dams of Danbred Yorkshire × Danbred Landrace, all inseminated with Danbred Duroc semen. They arrived at the experimental farm as weaners, and at ~30 kg of weight they were grouped across gender and size and moved to finisher pens. All pens were identical in dimensions (see Figure 1). Within each batch, the pens were randomly allocated to one level of three treatments in a 2 × 2 × 2 factorial design: (1) **TAIL**: pigs with undocked (*n* = 52 pens) vs. docked tails (*n* = 60 pens), (2) **STRAW**: not provided with straw (*n* = 56 pens) vs. provided with 150 g of straw per pig per day (*n* = 56 pens), (3) **STOCK**: stocking density of 0.73 m^2^/pig (*n* = 56 pens, 18 pigs per pen, high) vs. 1.21 m^2^/pig (*n* = 56 pens, 11 pigs per pen, low). The number of pens with each treatment combination within each batch can be seen in Table 1. In batch 2, less weaner pigs than planned arrived due to a serious tail damage outbreak, which especially affected the undocked pigs. Thus, less pens are included in batch 2 and only four pens in batch 2 had pigs with undocked tails (one with each treatment combination of STRAW and STOCK). Tail docking occurred according to Danish legislation within the first 4 days after birth with a hot-iron cutter to half of the tail's original length. Pigs with undocked and docked tails were housed separately from birth to slaughter. Pens including 18 pigs had one dry feeder with three feeding spaces, while pens including 11 pigs had one dry feeder with two feeding spaces. All feeding spaces were completely separated at the head and shoulder. Pigs were fed *ad libitum* with a commercial dry feed (15.1–15.6% crude protein), and the feeders were filled three times per day at 03:00 h, 10:00 h and 18:30 h. Artificial light was on from 05:30 to 18:30 h. The pigs were raised according to standard Danish practices and by educated stock people.

**Table 1:** Number of pens in total and for each batch, number of pens that were never scored with a tail damage event (“no tail damage” pens) and number of tail damage pairs within each treatment combination of docked and undocked pigs, with and without straw and with low (1.21 m^2^/pig) and high (0.73 m^2^/pig) stocking density.

	**Docked pigs**				**Undocked pigs**			
	**With straw**		**Without straw**		**With straw**		**Without straw**	
	**Low**	**High**	**Low**	**High**	**Low**	**High**	**Low**	**High**
No. pens in								
Batch 1, 3 and 4	4	4	4	4	4	4	4	4
Batch 2	3	3	3	3	1	1	1	1
Total	15	15	15	15	13	13	13	13
No. “no tail damage” pens	14	11	10	8	5	3	4	1
No. tail damage pairs	1	3	3	7	1	4	4	0

### Measurement of drinker use

**Figure 2:**
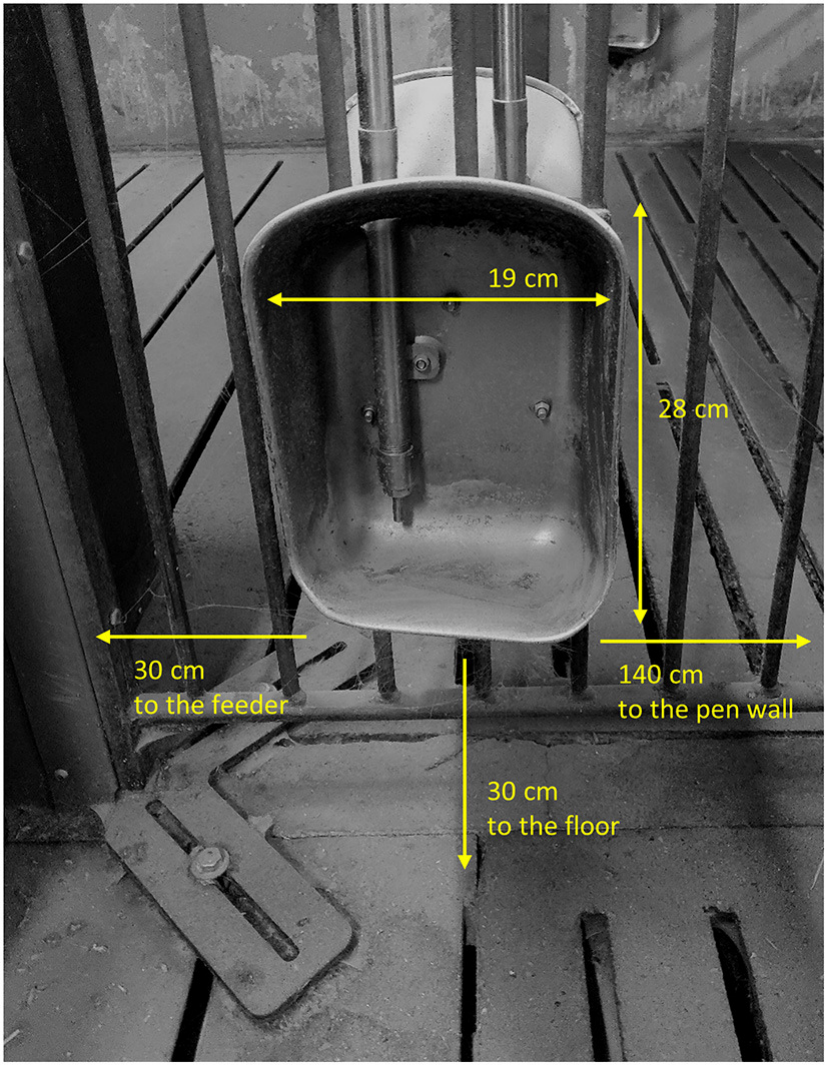
Design and dimensions of the drinking cups.

Each pen included two drinking cups: one located on the same side of the pen as the feeder (**F**), and one located on the opposite side of the pen as the feeder (**OF**; see Figure 1). The design and dimensions of the drinking cups can be seen in Figure 2. To investigate pigs' drinker use, each drinking cup included a separate liquid flow sensor (RS PRO Radialturbine Flowmåler, RS PRO, RS Components A/S, Copenhagen, Denmark, https://dk.rs-online.com/web/), and each sensor was connected to a system recording the pulses emitted from the sensor continuously every second. If no pulses were recorded within a specific second, the system noted this as a stop in water flow, and when pulses were again recorded by the system, this was noted as a start in water flow. From these recordings, the water use (L) and activation frequency (number of start recordings) per hour for each sensor were extracted. To calculate the water use from the number of pulses recorded, each sensor was calibrated by extracting a liter of water from the sensor with exact start and stop times of the extraction. This was repeated three times per sensor. The average number of pulses recorded within the time intervals (representing 1 L of water) was used to calculate the water use for each sensor throughout the study. To evaluate the accuracy over time, this calibration procedure was repeated a year later for half of the pens and sensors, reporting an inaccuracy of 8% or 80 ml per L of water used. In two pens, the sensors were broken, thus these two pens were not included in the data analysis (*n* = 110 pens; both pens with pigs with undocked tails, straw provided and high stocking density; one from batch 3 and one from batch 4). After descriptively analyzing water use on sensor level data from batch 1, 2 and 3 and finding that pigs often showed preference for one of the cups and sometimes changed this preference, the water cups were controlled for function and cleanliness in batch 4 from day 28 onwards on the same days as tail damage was scored inside the pen (see Scoring of tail damage events section).

### Scoring of tail damage events

Each day of the study period, the stock people recorded from outside the pen whether a pen had developed a tail damage event. Trained observers also recorded tail damage by more detailed observations each Monday, Wednesday and Friday by entering the pen, holding each tail between two fingers and scoring the tail according to the protocol presented in Wallgren et al. (12). A pen was recorded as having a tail damage event if and when at least one pig in the pen had a bleeding tail wound, based on either of the tail scorings. When a pig was observed with a bleeding tail wound, the pig was moved to a sickpen and the pen was no longer part of the study.

### Statistical analysis

All statistical analyses were performed in R Version 3.4.3 (13). All models were reduced using stepwise backward selection according to a 5% significance level (*P* < 0.05). All *post-hoc* analyses were performed using the “emmeans” package (14).

#### Data cleaning

Prior to analysis, the data on water use and activation frequency were cleaned. The purpose of the data cleaning was to ignore observation hours interpreted as outliers, arising from sensor errors such as either a stuck or leaking drinking nipple, resulting in too low or too high values. As the pigs often showed a preference for one of the two drinking cups in the pen, data were aggregated to include one measure per pen by taking the sum of the two sensors. Next, data were modeled separately for each batch through a Gaussian (normal) linear model accounting for both the age trend and the diurnal pattern in the water use/activation frequency (see details in the Effect of treatments on age trend and diurnal pattern section). From the model within each batch, the fitted values for each observation and the residual standard deviation for the full batch were extracted. Observation hours deviating more than four residual standard deviations from the fitted values of the model within each batch were ignored, ensuring that only greatly deviating outliers were not included. For water use data, this resulted in 0.5% (*n* = 1,016) of the observation hours being deleted from the data set and 172,322 observation hours remaining in the data set. For the activation frequency data, 0.8% (*n* = 1,392) of the observation hours were deleted, and 171,946 observation hours remained in the data set. The cleaned data were used in all analyses.

#### Age trend and diurnal pattern

The age trends and diurnal patterns of water use and activation frequency were modeled using data with one observation per pen and hour (the sum of the two sensors in each pen). This was done only on pens that were never scored with a tail damage event throughout the study period (*n* = 56, see Table 1). For both responses (L/pig/hour and activations/pig/hour), a linear mixed effect model [“glmmTMB” package (15)] assuming a Gaussian (normal) distribution was used, and both responses were square root transformed prior to analysis. The models specified a separate intercept for each day of the experiment nested within each pen and batch (1–4) and a first order autoregressive [AR(1)] covariance structure assuming a higher correlation between adjacent observation hours. The models accounted for the age trend in the water use and activation frequency by including the day of the experiment as a main effect. The models also accounted for the diurnal pattern by including the sum of three harmonic waves as this has previously been found to be the optimal number (16). A harmonic wave has the following general formula:


$$\begin{array}{l} Observation_{t}\;=\;Mean\;level+A\;\cdot \sin\left(b\cdot t+c\right) \end{array}$$

where *A* is the amplitude, *b* is the period and *c* is the phase shift. *A* and *c* for each of the three waves were estimated by the models, whereas *b* was provided to the models and represented waves with 24-h, 12-h and 8-h cycles. The models also included interactions between day of the experiment and the three harmonic waves to allow the amplitude of the waves to evolve with time. Table 2 presents the estimated values of the initial means and daily trends for water use and activation frequency as well as the estimated values of the amplitude and phase shift for the three harmonic waves describing the diurnal pattern.

**Table 2:** Estimated values (square root transformed) extracted from the model of the initial mean, daily trend, amplitude (*A*) and phase shift (*c*) of each wave summed to describe the diurnal pattern in water use and activation frequency in finisher pigs.

	**Water use (L/pig/hour)**			**Activation frequency (no/pig/hour)**		
Initial mean		0.343			1.31	
Trend (daily)		0.003214			0.000967	
**Wave estimates**	***A***	***b***	***c***	***A***	***b***	***c***
Wave 1	0.275	1•(2•π)/24	−1.84	0.82	1•(2•π)/24	−1.86
Wave 2	−0.0171	2•(2•π)/24	2.90	0.045	2•(2•π)/24	2.82
Wave 3	0.0896	3•(2•π)/24	1.67	0.27	3•(2•π)/24	1.59

The period (b) was provided to the model.

#### Effect of treatments on age trend and diurnal pattern

The effect of treatments on age trend and diurnal pattern in both water use and activation frequency were tested on data only including pens that were never scored with a tail damage event throughout the study period (*n* = 56, see Table 1). Gaussian (normal) linear mixed effects models [“glmmTMB” package (15)] were used with a first-order autoregressive AR(1) covariance structure. Prior to analysis, both responses (L/pig/hour and activations/pig/hour) were square root transformed. Also, the hours of day were divided into three time periods based on the diurnal pattern seen from the model described in section 2.4.2: (1) “Peak 1”: 06:00–11:59 h, (2) “Peak 2”: 12:00–17:59 h, (3) “Low”: 18:00–05:59 h. Both models (one for each response) included time period, week, TAIL, STRAW and STOCK as main effects as well as the interaction between time period and week and their two and three-way interactions to the treatments TAIL, STRAW and STOCK. The models further allowed for a separate intercept for each time period nested within week, pen, and batch. Besides the above, the effect of STOCK on the percentage of water use and activation frequency during the “Low” time period was tested using the non-parametric Mann-Whitney U test on data with one observation per pen (*n* = 56).

#### Drinking cup location

To investigate differences in the use of the two drinking cup locations per pen (F and OF, see Figure 1), data on sensor-level was used and the observation hours ignored through the data cleaning process described in Data cleaning section were also deleted from the sensor-level data set. This investigation was done only on pens that were never scored with a tail damage event throughout the study period (*n* = 56, see Table 1). The response variables (L/pig/hour and activations/pig/hour) could not be assumed to follow a Gaussian distribution. Thus, a non-parametric paired Wilcoxon test was used to compare the two drinking cup locations including a test across all batches, a test per batch and a test for each of the two stocking density treatments. Prior to the test, data were aggregated to one observation per pen per batch by taking the mean of all observations within each pen within each batch (*n* = 56 per cup location).

#### Changes prior to tail damage events

To investigate whether the daily level and diurnal pattern in water use and activation frequency changed prior to the recorded tail damage events, each “tail damage” pen was paired with a “no tail damage” pen that was not scored with a tail damage event prior to and at least 1 week after. The paired pens were from the same batch and had the same levels of the three treatments TAIL, STRAW and STOCK. If more than one “no tail damage” pens were available to be paired with a “tail damage” pen, the “no tail damage” pen in closest proximity to the “tail damage” pen was chosen. For each pair, only the last 3 days prior to the event and the day of the event (day-0) were included which resulted in 23 pairs with full data. See Table 1 for number of pairs within each treatment combination. Again, a time period variable was created (“Peak 1”: 06:00–11:59 h, “Peak 2”: 12:00–17:59 h, “Low”: 18:00–05:59 h). Four dependent variables per response (L/pig/hour and activations/pig/hour) were created for each time period and day: total sum, minimum, mean, and maximum. Each of these four variables were analyzed separately, but with similar models. Gaussian (normal) linear mixed effects models [“nlme” package (17)] were used with a first-order autoregressive AR(1) covariance structure. Prior to analysis, the mean, maximum and minimum of both responses were square root transformed. All models included pen type (no tail damage vs. tail damage pen), time period (Low, Peak 1, Peak 2), day (day-3, day-2, day-1 and day-0; categorical), age of the pigs at day-0 (continuous), and STOCK as main effects. All models further included the interactions between pen type, day, time period and STOCK. The models further allowed for a separate intercept for each day nested within pen and event number. Besides the above, the effect of pen type and day on the percentage of water use and activation frequency during the “Low” time period was tested using the non-parametric Mann-Whitney U test (pen type) and Kruskal-Wallis test (day) on data with one observation per pen type (*n* = 92) and day (*n* = 46).

## Results

### Age trend and diurnal pattern

**Figure 3:**
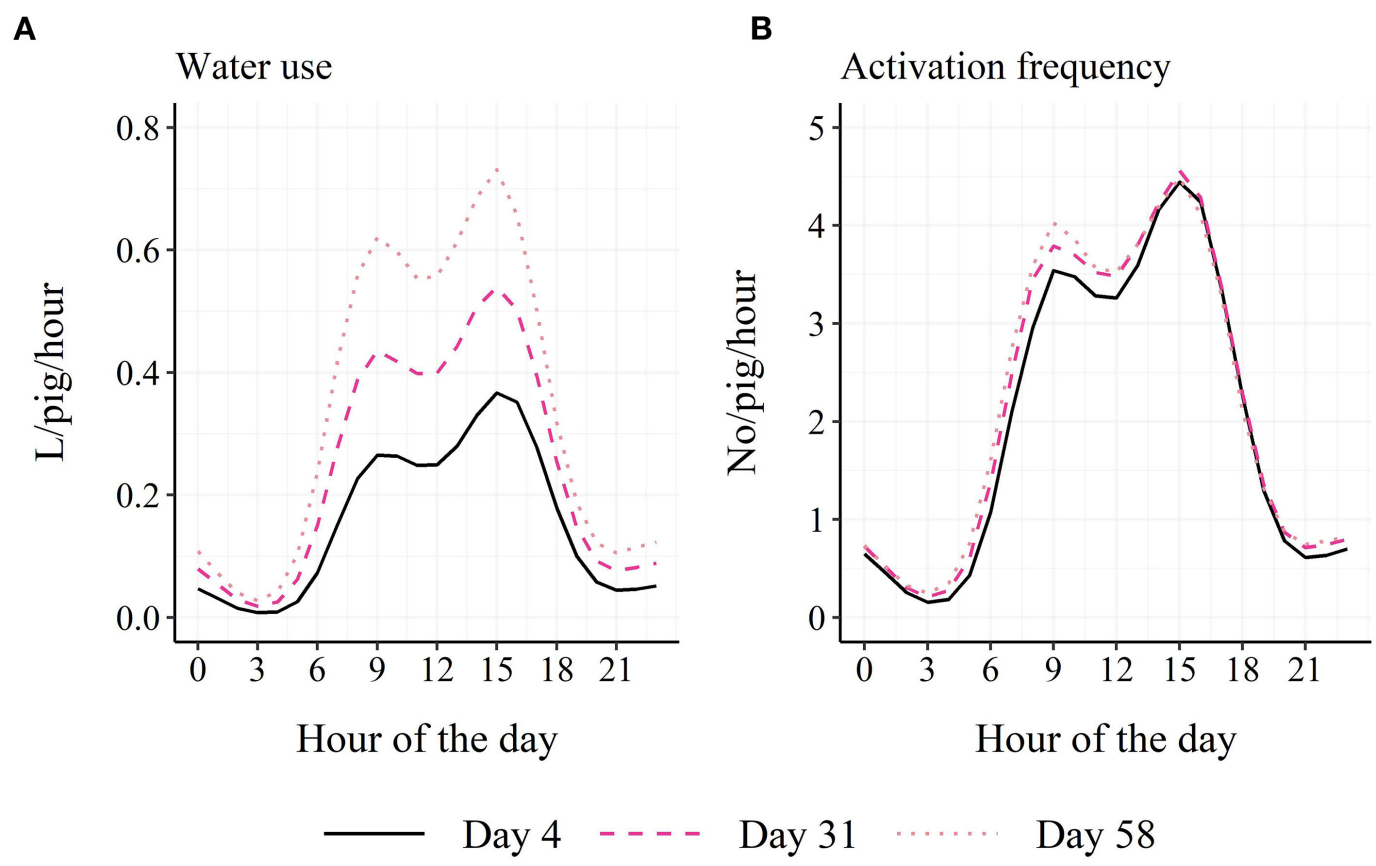
Model-estimated diurnal pattern in water use **(A)** and activation frequency **(B)** in finisher pigs on day 4, day 31 and day 58 in the finisher unit.

Based on model estimates, the finisher pigs used on average 5.9 L per pig and day with a range from 3.7 L in week 1 to 8.2 L in week 9 of the study period and activated the sensor on average 50 times per pig and day with a range from 48 activations in week 1 to 52 activations in week 9 of the study period. The fitted values of the models are graphically summarized in Figure 3, showing an increase in daily water use and a vertical parallel shift in the diurnal pattern in water use with days in the finisher unit. This development with days was not seen for activation frequency.

**Figure 4:**
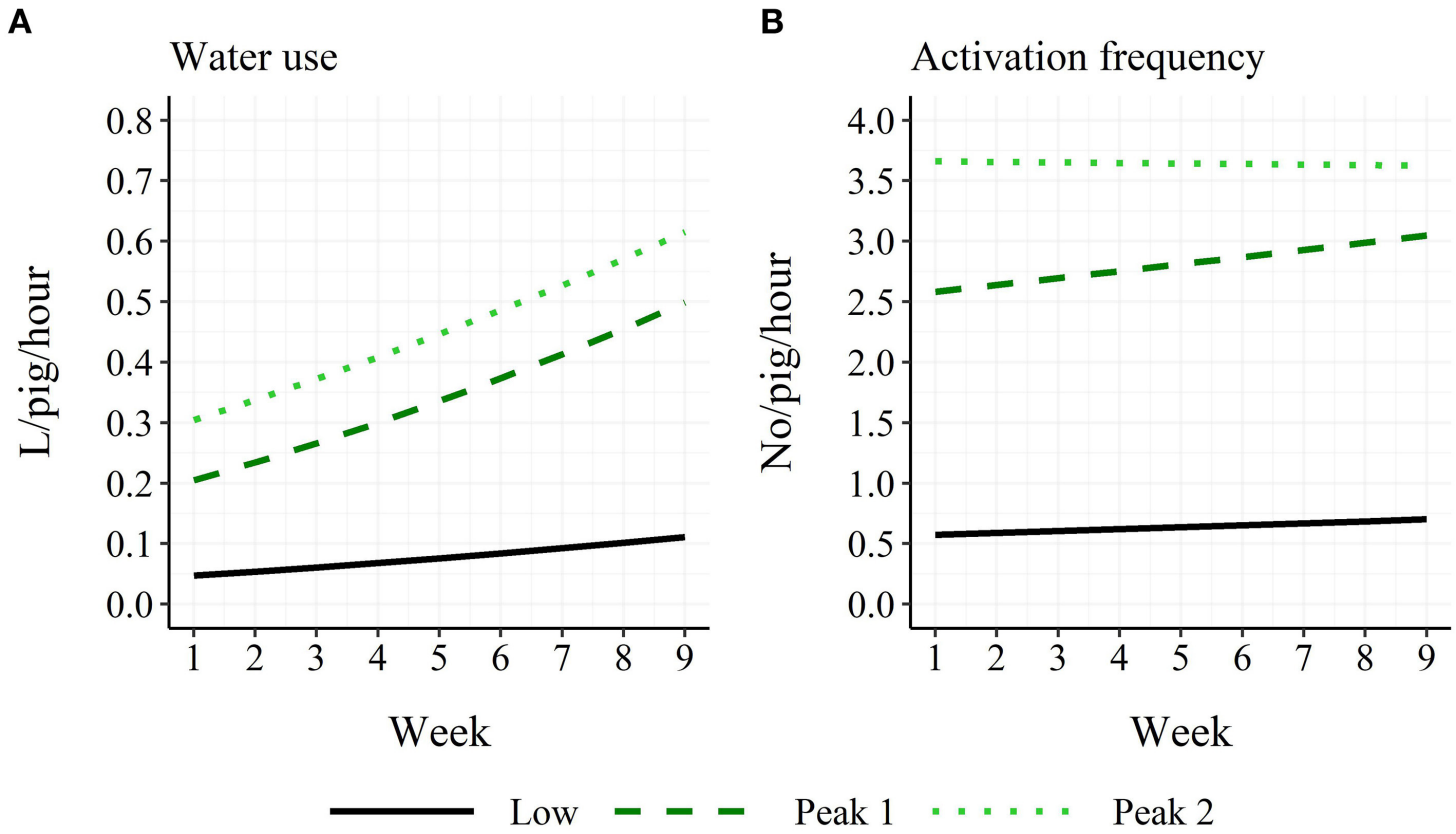
Age trend in model-estimated average hourly water use **(A)** and activation frequency **(B)** in finisher pigs for the three time periods: Low: 18:00–05:59 h; Peak 1: 06:00–11:59 h; Peak 2: 12:00–17:59 h.

A two-way interaction was found between time period and week for both water use ($\chi_{2}^{2}$ = 127.2; *P* < 0.001) and activation frequency ($\chi_{2}^{2}$ = 18.6; *P* < 0.001). For water use, all three time periods differed in their intercepts at week 1 (“Low”: 0.047 L/pig/hour; “Peak 1”: 0.205 L/pig/hour; “Peak 2”: 0.304 L/pig/hour), while the “Peak 1” and “Peak 2” time periods had higher slopes than the “Low” time period (before back-transformation: “Low”: 0.015; “Peak 1”: 0.032; “Peak 2”: 0.029; Figure 4A). For activation frequency, all three time periods differed in their intercepts (“Low”: 0.58 no/pig/hour; “Peak 1”: 2.58 no/pig/hour; “Peak 2”: 3.66 no/pig/hour), while the “Peak 2” time period had a lower slope than the “Peak 1” time period (before back-transformation: “Low”: 0.0099; “Peak 1”: 0.017; “Peak 2”: −0.0011; Figure 4B).

### Effect of treatments on age trend and diurnal pattern

**Figure 5:**
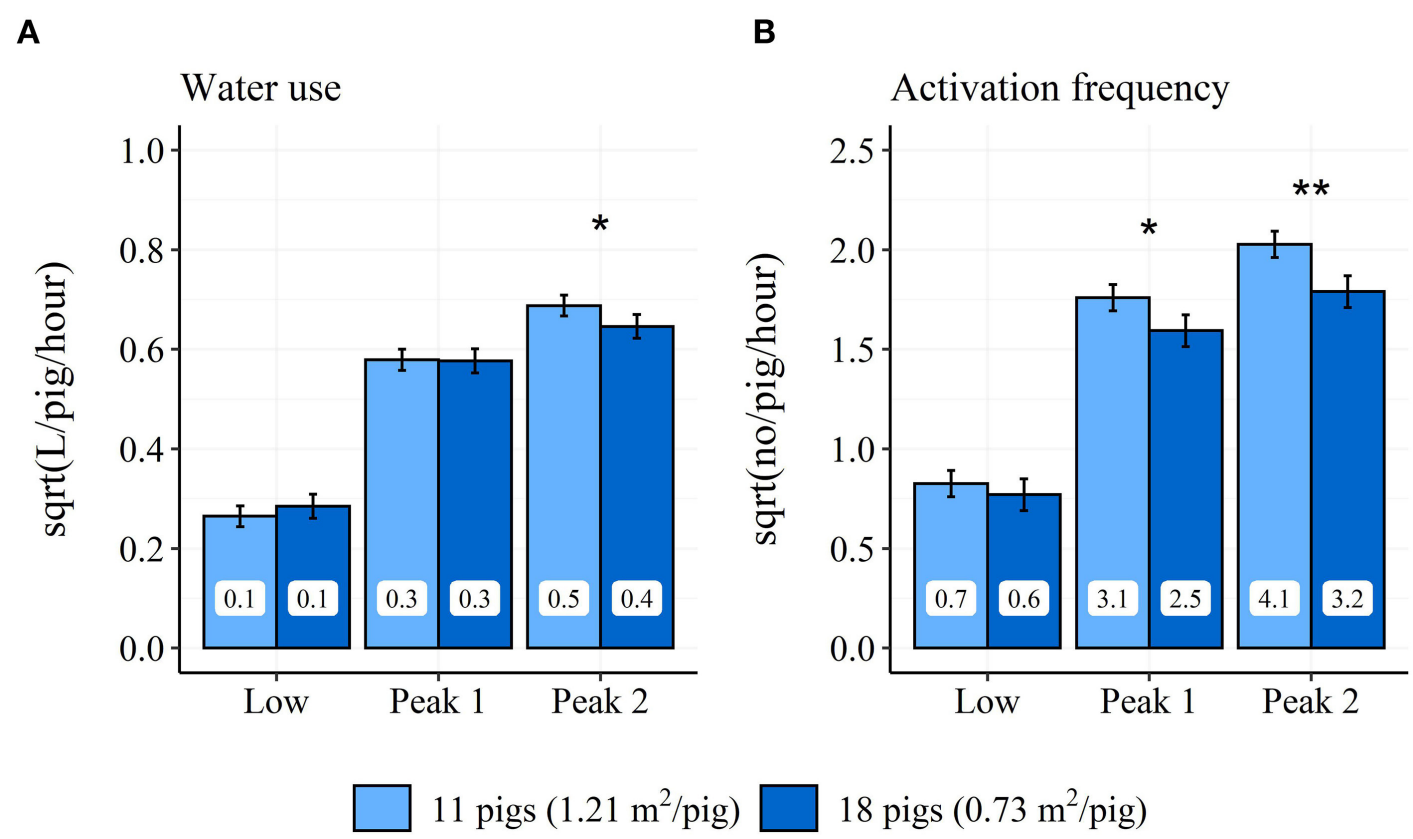
Differences in model estimated average hourly water use **(A)** and activation frequency **(B)** between pens with low and high stocking density during the “Low” (18:00–05:59 h), “Peak 1” (06:00–11:59 h), and “Peak 2” (12:00–17:59 h) time periods across the finisher period. Error-bars represent standard errors of the mean. The back-transformed model estimates are shown within each bar. *P < 0.5, **P < 0.01 between pens with high and low stocking density.

Only the treatment STOCK affected the diurnal pattern of both water use and activation frequency through a two-way interaction between time period and STOCK (water use: $\chi_{2}^{2}$ = 72.7, *P* < 0.001; activation frequency: $\chi_{2}^{2}$ = 98.9, *P* < 0.001). Pens with the low stocking density had a 0.55 higher number of activations per pig and hour (3.3 activations/pig for the entire time period) in the “Peak 1” time period compared to pens with the high stocking density. Further, pens with a low stocking density used 0.056 L more water per pig and hour (0.336 L/pig for the entire time period) and had a 0.91 higher number of activations per pig and hour (5.46 activations/pig for the entire time period) in the “Peak 2” time period compared to pens with the high stocking density (see Figure 5). STOCK also showed a tendency to affect the percentage of water use (*P* = 0.07) and activation frequency (*P* = 0.08) during the “Low” time period. During the “Low” time period, pens with the low stocking density used 24% of their water and activations, whereas pens with the high stocking density used 27% of their water and 26% of their activations.

### Drinking cup location

**Figure 6:**
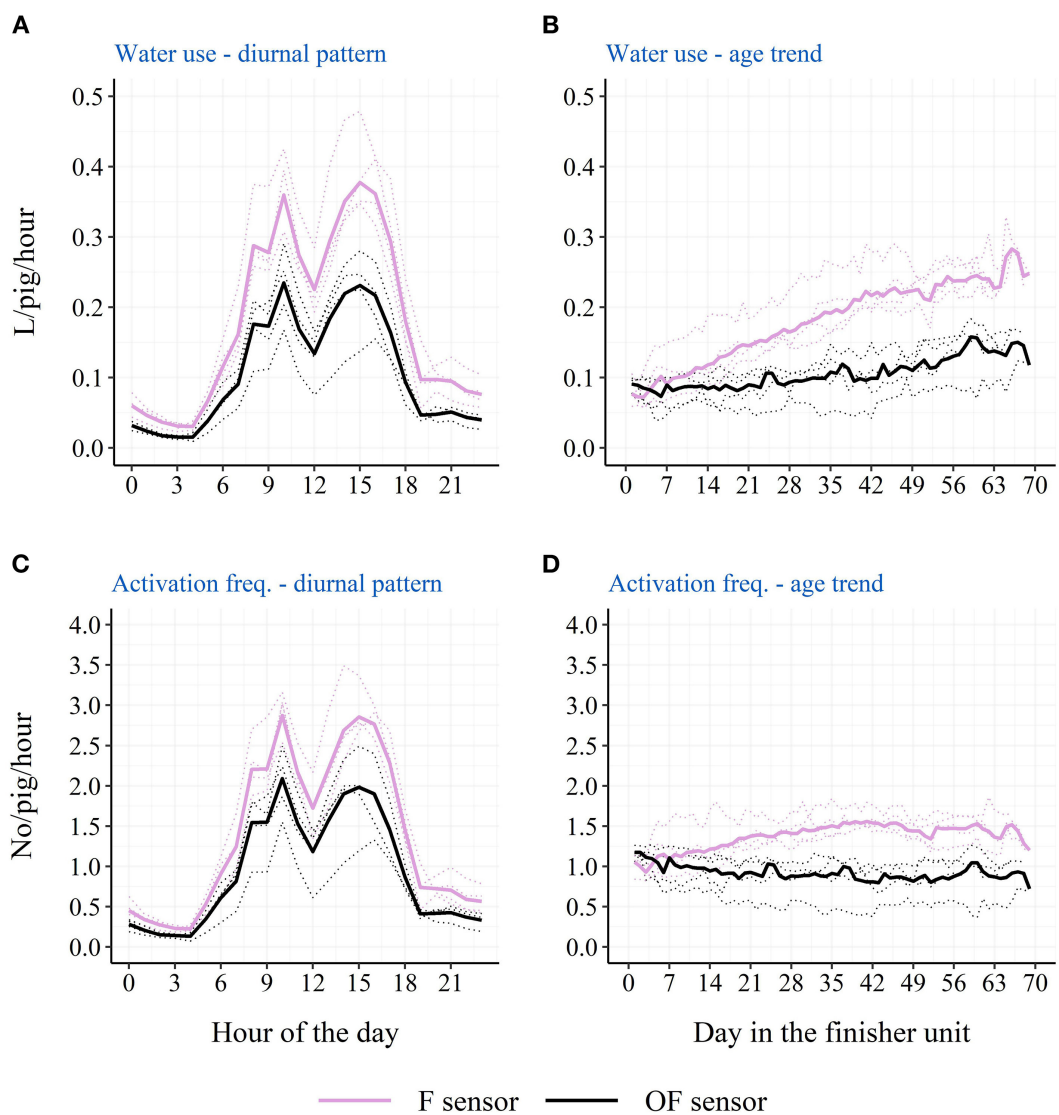
Descriptive diurnal pattern **(A)** and age trend **(B)** in the water use in finisher pigs as well as the diurnal pattern **(C)** and age trend **(D)** in the activation frequency in finisher pigs for the two drinking cup sensor locations: F sensor (on the same side of the pen as the feeder) and OF sensor (on the opposite side of the pen as the feeder). Dotted lines represent each single batch, while solid lines represent the average across the four batches.

**Figure 7:**
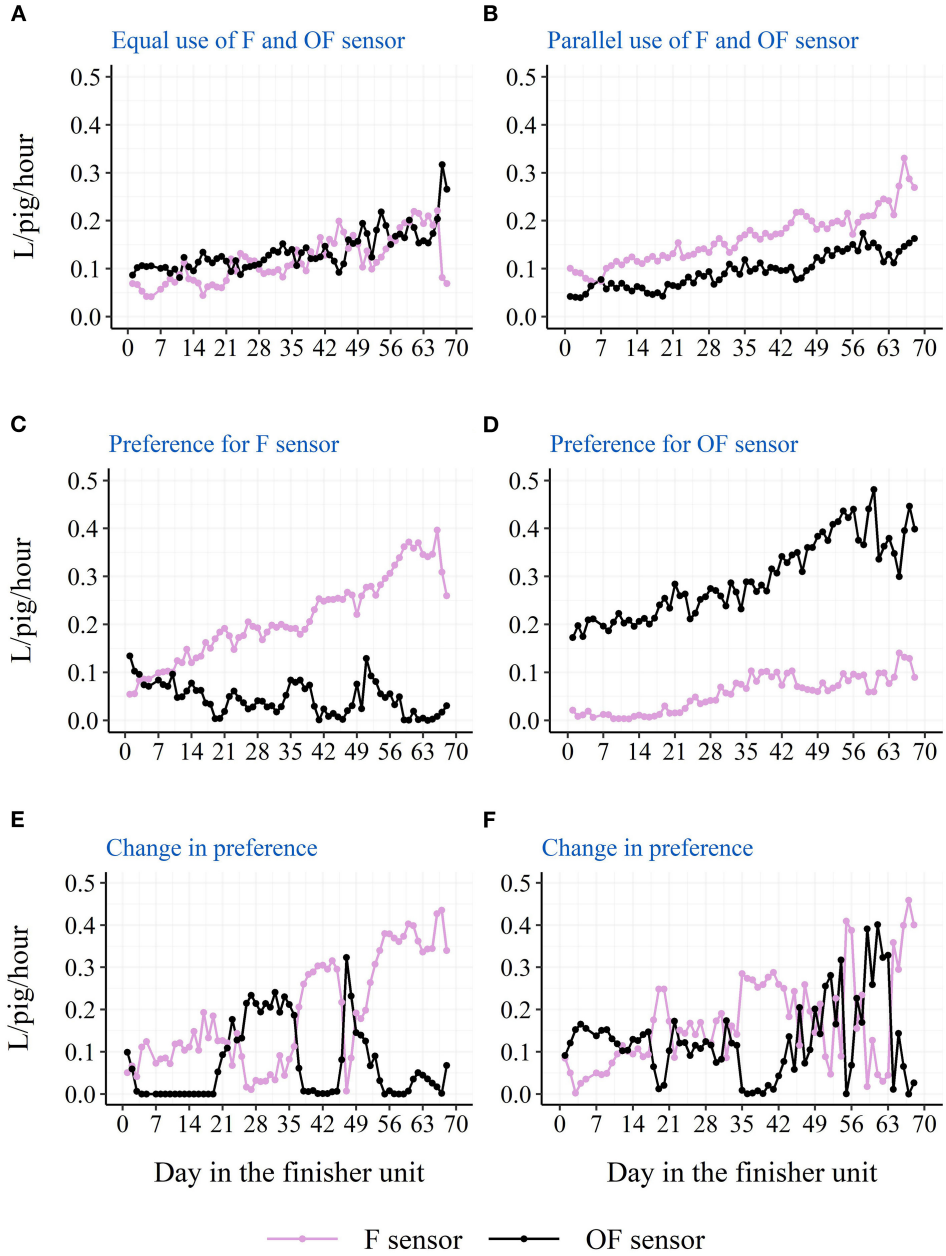
Examples from batch 4 of single pens variations in raw average hourly water use from the two drinking cups (F sensor: at the same side of the pen as the feeder; OF sensor: on the opposite side of the pen as the feeder). **(A)** Docked tails, without straw, high stocking density. **(B)** Docked tails, with straw, low stocking density. **(C,D)** Docked tails, without straw, low stocking density. **(E,F)** Docked tails, with straw, high stocking density.

**Figure 8:**
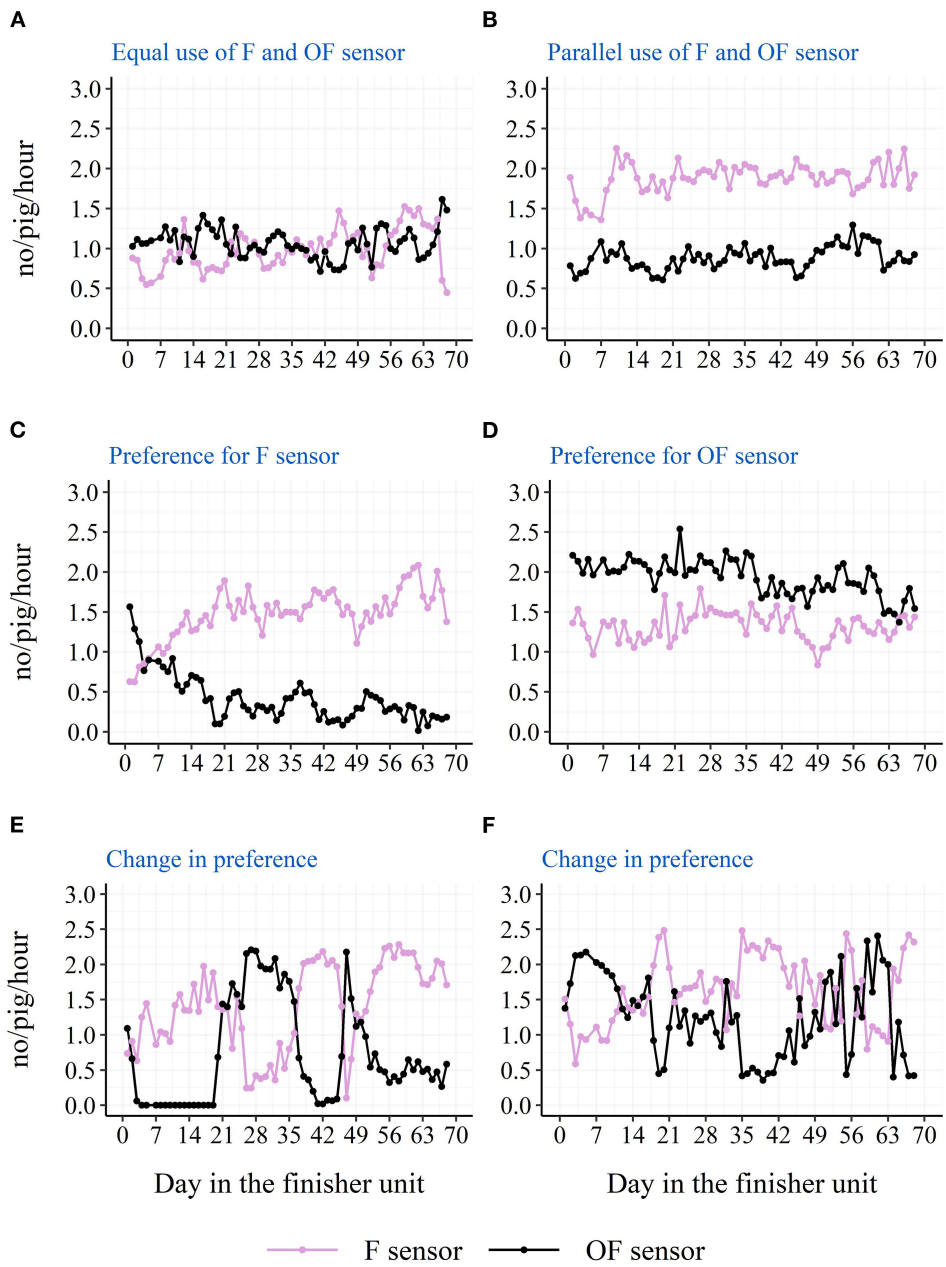
Examples from batch 4 of single pens variations in raw average hourly activations of the two drinking cups (F sensor: at the same side of the pen as the feeder; OF sensor: on the opposite side of the pen as the feeder). **(A)** Docked tails, without straw, high stocking density. **(B)** Docked tails, with straw, low stocking density. **(C,D)** Docked tails, without straw, low stocking density. **(E,F)** Docked tails, with straw, high stocking density.

The descriptive diurnal pattern and age trend in water use and activation frequency for each of the two cup locations can be seen in Figure 6. The F cup had an overall higher water use and activation frequency than the OF cup (Table 3). When testing each batch separately, this was only the case for batch 1 and batch 2, although numerically in the same direction for batch 3 and batch 4 (Table 3). When testing each STOCK treatment separately, this was only the case for pens with the low stocking density, although numerically in the same direction for pens with the high stocking density. When investigating the age trend for each individual pen descriptively, the pens showed very different patterns in their use of the two drinking cups, e.g., some pens preferred one cup location over the other, whereas other pens changed their preference during the study period. Figures 7, 8 shows examples of these patterns. These examples are from batch 4 where the cleanliness and function of the water cups were controlled from day 28 and onwards. The water cups always functioned. For the examples in Figures 7, 8A,B,D,E, no cleaning of the water cups were done. For Figures 7, 8C, the OF water cup was cleaned on day 44 and 65. For Figures 7, 8F, the OF water cup was cleaned on day 42.

**Table 3:** The mean water use and activation frequency for the sensor located at the same side of the pen as the feeder (F) and for the sensor located on the opposite side of the pen as the feeder (OF), across all batches, for each batch and for each stocking density treatment (High: 0.73 m^2^/pig, 18 pigs; Low: 1.21 m^2^/pig, 11 pigs).

		**Water use (L/pig/hour)**				**Activation frequency (no/pig/hour)**			
	**N**	**F**	**OF**	**Diff**	***P***	**F**	**OF**	**Diff**	***P***
All batches	56	0.179	0.104	0.075	< 0.001	1.38	0.92	0.46	< 0.001
Batch									
1	13	0.183	0.070	0.113	0.01	1.29	0.59	0.70	0.01
2	10	0.225	0.095	0.130	0.05	1.67	0.90	0.77	0.05
3	14	0.164	0.119	0.045	0.20	1.33	1.01	0.32	0.20
4	19	0.164	0.120	0.044	0.20	1.33	1.09	0.24	0.20
Stocking density									
High	23	0.160	0.118	0.041	0.20	1.19	0.94	0.26	0.20
Low	33	0.193	0.094	0.099	< 0.001	1.51	0.91	0.60	< 0.001

N, number of pens.

### Changes prior to tail damage events

**Figure 9:**
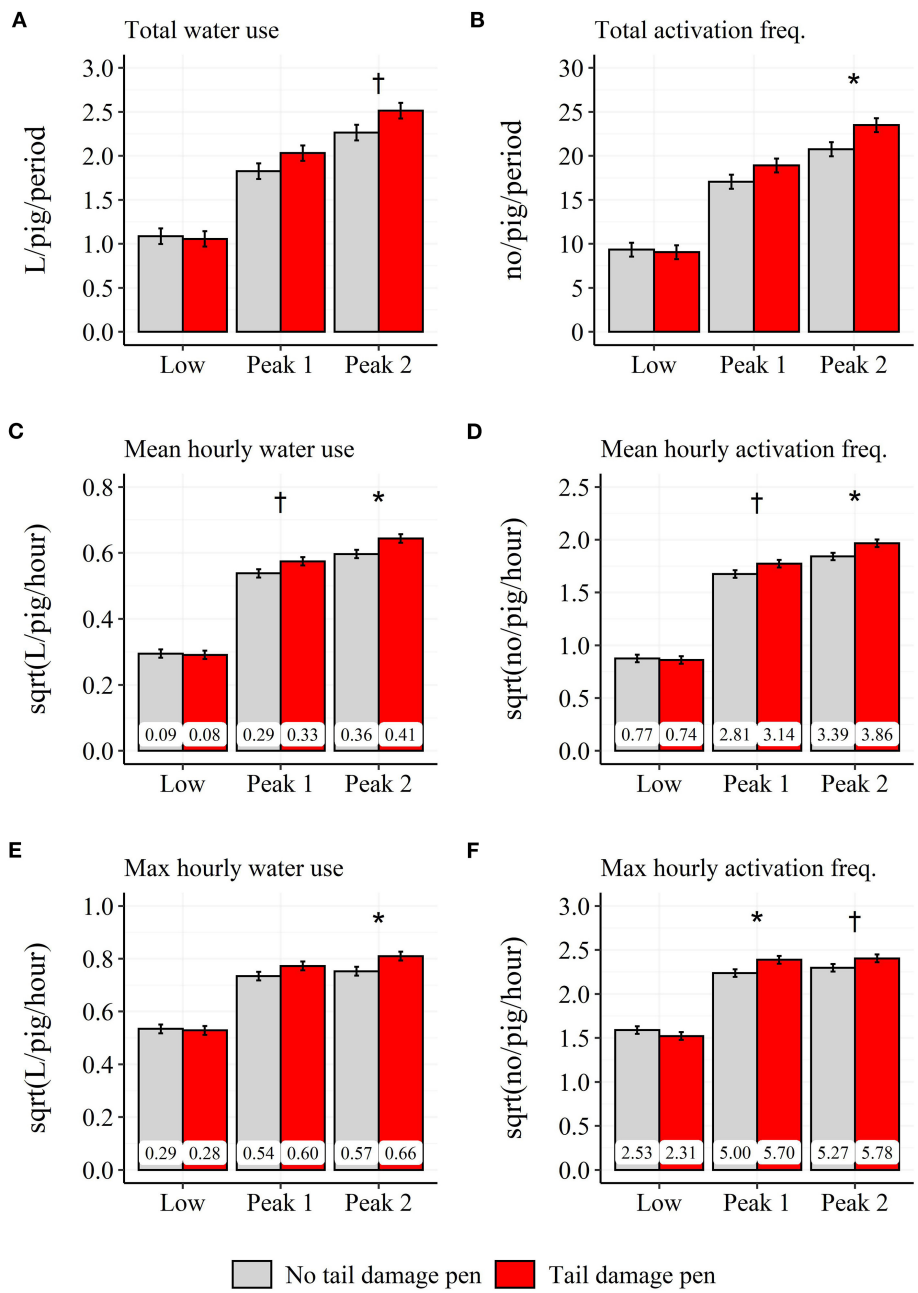
Difference between pens with and without tail damage across the 3 days prior to and the day of the tail damage event in model-estimated daily total **(A,B)**, mean-hourly **(C,D)** and max hourly **(E,F)**, water use **(A,C,E)** and activation frequency **(B,D,F)** during the three time periods: Low: 18:00–05:59 h; Peak 1: 06:00–11:59 h; Peak 2: 12:00–17:59 h. Error-bars represent standard errors of the mean. The back-transformed model estimates are shown within each bar. ^†^*P* < 0.1, *P < 0.05 between pens with and without tail damage.

A two-way interaction was found between pen type and time period for the total ($\chi_{2}^{2}$ = 9.8; *P* < 0.01), mean ($\chi_{2}^{2}$ = 19.0; *P* < 0.001) and maximum water use ($\chi_{2}^{2}$ = 13.3; *P* < 0.01), independent of day relative to the tail damage event (see Figures 9A,C,E). During “Peak 1,” pens with a tail damage event used 0.041 L more water per pig and hour compared to pens without a tail damage event. During the “Peak 2” time period, pens with a tail damage event used 0.058 L more water per pig and hour, 0.240 L more water per pig across the entire time period and had a higher maximum in water use compared to pens without a tail damage event (Pens with tail damage: 0.656 L/pig/hour; Pens without tail damage: 0.567 L/pig/hour).

A two-way interaction was found between pen type and time period for the total ($\chi_{2}^{2}$ = 20.4; *P* < 0.001), mean ($\chi_{2}^{2}$ = 20.9; *P* < 0.001) and maximum activation frequency ($\chi_{2}^{2}$ = 22.2; *P* < 0.001), independent of day relative to the tail damage event (see Figures 9B,D,F). During the “Peak 1” time period, pens with a tail damage event had a 0.34 higher number of activations per pig and hour and a higher maximum in number of activations compared to pens without a tail damage event (Pens with tail damage: 5.70 no/pig/hour; Pens without tail damage: 5.00 no/pig/hour). During the “Peak 2” time period, pens with a tail damage event had a 0.47 higher number of activations per pig and hour, a 3.21 higher number of activations per pig across the entire time period and a higher maximum in number of activations compared to pens without a tail damage event (Pens with tail damage: 5.78 no/pig/hour; Pens without tail damage: 5.27 no/pig/hour).

The percentage of water use and activation frequency during the “Low” time period was not affected by either pen type or day prior to the event.

## Discussion

### Age trend and diurnal pattern

The water use showed a linear increase from ~4 to 8 L per pig and day during the finisher period, and this increase was mainly seen due to an increase in the water use within the two peaks of the diurnal pattern from 06:00 h to 18:00 h. Such an increase with time was not seen for activation frequency with an average of 50 activations per pig and day. This argues that the pigs either drink more per visit as they get older, that they learn to drink from the cup without stopping their pressure to the nipple or that they use the drinking nipple less for general manipulation as they get older. Andersen et al. (3), comparing similar group sizes and stocking densities as the current study (14 vs. 18 pigs per pen; 0.94 vs. 0.73 m^2^/pig) found similar water use by pigs during the first 4 weeks of the finisher period of 4.5 L per pig and day, while Andersen et al. (4) found a daily water use of 4.99 L per weaner pig. According to the increase with age seen in the current study, the water use of weaners should be below 4 L per day and pig. Andersen et al. (4) used smaller group sizes and lower stocking densities (3 vs. 10 pigs per pen and drinking nipple; 3.1 vs. 1.0 m^2^/pig), arguing that if space is available, the pigs can and will use more water than what is seen under normal production practices. However, Turner et al. (5) saw a larger water use per day and pig in finisher pens with the larger group size (60 vs. 20 pigs per pen; similar stocking densities), while pigs in pens with the smaller group size and with the more generous drinker allocation (one drinker per 10 vs. 20 pigs) visited the drinker more often and drank for longer per day. Similar results were found for pigs' eating behavior (18) and is probably seen due to less social stress with fewer pigs and higher drinker/feeder allocation. Thus, the water use of pigs does not necessarily reflect the duration or frequency of pigs' drinking behavior. Misra et al. (6) found an average water use across the finisher period of 10–11 L per pig and day. However, in their study pigs had access to water both from a drinking bowl and a nipple above the feed trough, making it possible for the pigs to mix the water with the feed. This difference between the two studies may explain the lower average water use in the current study.

Both the water use and activation frequency showed a diurnal pattern with two peaks during the day time, which fit the general assumption that pigs rest during the night hours and are more active during daylight hours (19). This pattern in water use is similar to what was found by previous studies (16, 20) with small differences in the timing of the peaks. Jensen et al. (8) and Misra et al. (6) only saw one large peak, but within the same hours as the two peaks found in the current study. Villagrá et al. (21) found two peaks in the diurnal drinking pattern of pigs with different timing both compared to the current study and when comparing the two farms included in their study. The above illustrates the effects that different on-farm conditions can have on pigs' drinking behavior and the importance of modeling the drinking pattern individually for the single herd if using this behavior as an indicator of for example pig welfare, stress, activity or feeding behavior, or as an early detector of undesirable events such as tail damage.

### Effect of treatments

Villagrá et al. (21) argued that the differences in diurnal drinking pattern between farms could be due to differences in the ventilation system and thereby in the room temperature. Also, both Andersen et al. (4) and Andersen et al. (3) found differences not at a daily level of water use but in the diurnal drinking pattern of weaners and finishers between pens with low and high stocking density. Thus, the experiences of stressors and different environmental conditions could be reflected in a change of the diurnal drinking pattern. However, in the current study, neither an undocked tail nor lack of straw as enrichment caused a change in either the age trend or the diurnal pattern of water use and activation frequency. When provided with straw, the pigs may eat the straw, thereby become thirstier and drink more. On the other hand, when not provided with straw, the pigs could use the drinking nipple as a manipulation device. Both situations will increase the water use and activation frequency and could be the reason for why no difference was seen between pens with and without straw. Misra et al. (6) found that the addition of more proper enrichment (addition of fresh grass to a wooden post and a hanging rubber toy) decreased the water used and wasted, indicating the pigs may have used the drinker less for redirected manipulative behavior when more proper enrichment was added (6). *Post-hoc* analysis showed that this effect of enrichment was only seen in pens with the high group size of 48 pigs, whereas only a tendency was found with 24 pigs and no effect with 12 pigs (same ratio of drinkers and space per pig) (6). Thus, to see a sufficient decrease in redirected manipulative behavior toward the drinkers, a larger group size or enrichment with higher value than used in the current study may be needed. Also, it may be that the intake of fresh grass compared to straw make the pigs less thirsty, as discussed above.

One factor that affected the diurnal pattern of both water use and activation frequency in the current study was stocking density. The current study found a higher activation frequency in pens with the low stocking density during the two peaks in the diurnal pattern as well as a higher water use during the second peak in the diurnal pattern. Furthermore, the percentage of water used and activations of the drinking cup during the low activity period tended to be higher in pens with the high stocking density, similar to a previous study (3). At last, the pigs in pens with the low stocking density showed a preference for the drinking cup on the same side of the pen as the feeder; a preference that was less pronounced at the high stocking density. The results argue that pigs in pens with the low stocking density may have better access to the drinking cups during their normal period of high activity (22). This suggests that the access to the drinking cups could be restricted in pens with the high stocking density; although a stocking density of 0.73 m^2^/pig is within the legislative space demand for pigs up to 110 kg (0.65 m^2^/pig; EU Council Directive 2008/120/EC). Both stocking density treatments also fulfill the Danish recommendations for pig:drinker ratio (no higher than 10:1) by being < 6:1 within the low stocking density treatment and 9:1 within the high stocking density treatment. When dividing the day into 8-h periods, Andersen et al. (4) saw a higher water use during the afternoon (14:00–22:00 h) and night (22:00–06:00 h) in the weaner pens with the high stocking density, whereas the pigs in the weaner pens with the low stocking density used more water during the morning (06:00–14:00 h). When dividing the day into 12-h periods representing day and night, Andersen et al. (3) saw a higher proportion of water consumed during the night in the finisher pens with the high stocking density. Andersen et al. (4) used similar pig:drinker ratios but lower group sizes and thereby lower stocking density than the current study, whereas Andersen et al. (3) used similar pig:drinker ratios, group sizes and stocking densities as the current study. All three studies showed that differences in the stocking density, group size or pigs:drinker ratio reflected in a change in the pigs' diurnal drinking behavior. It confirms the need to consider stocking density, group size and pig:drinker ratio when studying pigs' drinking behavior or using it for other purposes in the produciton of pigs. Although, Misra et al. (6) found no effect of group size variations of 12, 24 and 48 pigs on pigs use of water, arguing that stocking density and/or pigs:drinker ratio may be more important factors when considering pigs' use of drinkers. The combined results suggest that the recommended pig:drinker ratio of 10:1 is limiting the pigs in their access to water, meaning that there ideally should be fewer pigs per drinker in a production setting.

### Drinking cup location

Location of the drinking cup also seemed to affect the drinking behavior of the pigs with a higher water use and activation frequency on the drinking cup located next to the feeder. Also here, this was mainly seen due to a higher use of the drinking cup during the two large peaks of the diurnal pattern. As pigs' eating and drinking patterns seem to follow each other (21), and 75% of the pigs' water intake has been shown to be associated with the pigs' feed intake (23), one explanation could be that the pigs eat and then visit the nearest cup for drinking. Thus, the nearest drinking cup may be the pigs' preferred cup to use, and they will turn to the cup on the other side of the pen when the preferred cup is not available. If only one drinking cup is available in the pen due to for example a smaller group size, the results of the current study suggest that this cup should be placed next to the feeder. However, the current study cannot elucidate on whether it would be an advantage to have both cups on the same side of the pen close to the feeder, or whether it is better to separate them as in the current study to ensure more space around the cups. It is also important to remember that the above results are based on averages across pens and the finisher period. Some pens preferred the cup on the opposite side of the pen as the feeder, and some pens even changed their preference during the finisher period. The latter could be due to a sudden malfunction of one of the drinking nipples or due to a contamination of one of the drinking cups, e.g., with feces, which further confirms the need of more than one drinker per pen independent of the stocking density and number of pigs. Although, the examples presented from batch 4 in Figures 7, 8 do not confirm sudden malfunction or contamination to be the sole reasons. Perhaps monitoring water use at sensor level could be used to intervene as soon as possible in such situations to avoid a limited water access for the pigs. To our knowledge, no previous study has investigated the effect of drinker location on pigs' drinking behavior.

### Tail damage

General differences (the same for all 4 days) were seen between pens with and without a tail damage event in both water use and activation frequency. This was seen only during the two peaks of the diurnal patterns with higher water use and activation frequency in the pens with a tail damage event, and may be a sign of general higher activity in the pen, an indicator that may also change prior to tail damage [e.g., (24, 25)]. Previous studies on prediction of tail damage events found promising predictive value in finisher pigs' drinker use in relation to tail damage (7, 9). The differences in water use and activation frequency found in the current study may have been part of the classification of pens into tail damage pens and no tail damage pens, especially as the study of Larsen et al. (7) did include mean and maximum (among others) of both water use and activation frequency as predictors within the last 3 days prior to the event (7). When tested in a real-life setting, the prediction model produced too many false alarms of tail damage and the authors concluded that pigs drinker use was not a tail damage specific indicator (7). In the current study, no changes in either water use or activation frequency was seen the last 3 days prior to an event of tail damage. Thus, it is still unclear when these differences between pens with and without tail damage arise. Due to data availability, it was unfortunately not possible to include more days prior to tail damage in the current study. A limitation of the current study and the previously developed prediction models of tail damage is that drinker use is monitored and tail damage is scored on pen level. Drinker use may be a more specific indicator if monitored on pig level to e.g., identify the tail biter or the pig with a bitten tail, as pronounced tail biters and victims have been shown to differ in and change their behavior prior to the observation of tail damage (26–28). A method to obtain such individual pig registrations of pigs water use could be to combine the water flow-meter recordings with RFID technology (29).

## Conclusion

The water use of pigs increased from around 3.7–8.2 L per pig and day during a finisher period of 9 weeks, and this increase was mainly seen during the two large peaks of the diurnal pattern within the pigs' active period. No such increase was seen in the activation frequency at around 50 activations per pig and day. Neither water use nor activation frequency was affected by whether a pen had pigs with undocked or docked tails, or whether it was provided with straw or not. However, a decrease in stocking density increased both water use and activation frequency during the active period, suggesting that pigs at the standard space allowance and with pig:drinker ratio within the recommendations could be restricted in their access to the drinking cups. The pigs also seemed to prefer to use the drinking cup closest to the feeder, although the descriptive analysis showed that some pens had opposite preferences and even changed their preferences during the study period. Water use and activation frequency did not change the last 3 days prior to an event of tail damage, but general differences were seen between pens with and without a tail damage event during the two large peaks of the diurnal pattern in both water use and activation frequency. The current results may explain the success of previous studies in classifying tail damage pens from pens without tail damage using sensor data on drinker use.

## Data availability statement

The raw data supporting the conclusions of this article will be made available by the authors, without undue reservation.

## Ethics statement

The animal study was reviewed and approved by Danish Animal Experiments Inspectorate (Journal No. 2015-15-0201-00593).

## Author contributions

ML and LP contributed to the design and conduction of the study. LP ensured the funding for the study. ML performed the literature study, the descriptive and inferential analyses, and wrote the first draft of the manuscript. LP supervised ML throughout the production of the draft. Both authors contributed to manuscript revision and have read and approved the submitted version.

## Funding

This research was funded by the Green Development and Demonstration Programme under the Ministry of Food, Agriculture and Fisheries, Denmark, Project IntactTails (J. Nr. 34009-13-0743).

## Conflict of interest

The authors declare that the research was conducted in the absence of any commercial or financial relationships that could be construed as a potential conflict of interest.

## Publisher's note

All claims expressed in this article are solely those of the authors and do not necessarily represent those of their affiliated organizations, or those of the publisher, the editors and the reviewers. Any product that may be evaluated in this article, or claim that may be made by its manufacturer, is not guaranteed or endorsed by the publisher.
